# Karyotype Abnormalities in the X Chromosome Predict Response to the Growth Hormone Therapy in Turner Syndrome

**DOI:** 10.3390/jcm10215076

**Published:** 2021-10-29

**Authors:** Jakub Kasprzyk, Marcin Włodarczyk, Aleksandra Sobolewska-Włodarczyk, Katarzyna Wieczorek-Szukała, Renata Stawerska, Maciej Hilczer, Andrzej Lewiński

**Affiliations:** 1 Drewnica Masovian Voivodship Hospital, 05-091 Ząbki, Poland; jkbkasprzyk@gmail.com; 2 Department of General and Oncological Surgery, Medical University of Lodz, 92-213 Lodz, Poland; marcin.wlodarczyk@umed.lodz.pl; 3 Department of Gastroenterology, Medial University of Lodz, 92-213 Lodz, Poland; Aleksandra.sobolewska-wlodarczyk@umed.lodz.pl; 4 Department of Endocrinology and Metabolic Diseases, Medical University of Lodz, 93-338 Lodz, Poland; katarzyna.wieczorek@umed.lodz.pl; 5 Department of Pediatric Endocrinology, Medical University of Lodz, 93-338 Lodz, Poland; renata.stawerska@umed.lodz.pl; 6 Department of Endocrinology and Metabolic Diseases, Polish Mother’s Memorial Hospital–Research Institute, 93-338 Lodz, Poland; maciej.hilczer@umed.lodz.pl

**Keywords:** turner syndrome, growth hormone therapy, karyotype abnormalities

## Abstract

Short stature is characteristic for Turner syndrome (TS) patients, and particular karyotype abnormalities of the X chromosome may be associated with different responsiveness to recombinant human GH (rhGH) therapy. The aim of the study was to analyze the effect of different types of TS karyotype abnormalities on the response to rhGH therapy. A total of 57 prepubertal patients with TS treated with rhGH with a 3 year follow-up were enrolled in the study and categorized according to their karyotype as X monosomy (*n* = 35), isochromosome (*n* = 11), marker chromosome (*n* = 5), or X-mosaicism (*n* = 6). Height and height velocity (HV) were evaluated annually. In the first year, all groups responded well to the therapy. In the second year, HV deteriorated significantly in X-monosomy and isochromosome in comparison to the remaining two groups (*p* = 0.0007). After 3 years of therapy, all patients improved the score in comparison to their target height, but better outcomes were achieved in patients with marker chromosome and X-mosaicism (*p* = 0.0072). X-monosomy or isochromosome determined a poorer response during the second and third year of rhGH therapy. The results of the study indicate that the effects of rhGH therapy in patients with TS may depend on the type of TS karyotype causing the syndrome.

## 1. Introduction

Turner syndrome (TS) occurs in approximately 1:2500 live born females, which makes it one of the most common chromosome abnormalities [1]. The complete or partial absence of one of the X chromosomes is responsible for the clinical symptoms of the patients. Short stature, gonadal dysgenesis, congenital defects of aorta, heart, or kidney, skeletal abnormalities, and various dysmorphic features, such as webbed neck, low hairline, broad chest, widely spaced nipples, and shortened metacarpal IV, occur in affected individuals [2,3].

However, the observed manifestations of TS may vary according to the range of chromosomal aberration. More than half of TS children present X-monosomy, and 5–10% of the cases result from a duplication of the long arm of the X-chromosome (isochromosome), while the remaining patients present karyotype mosaicism with two or more different cellular lines [1,4].

Short stature is one of the most characteristic features of TS patients. The mean final height of TS patients who do not undergo any therapy is around 140–145 cm, which is more than 2.0 SD lower than the final height of the population of healthy women. It is also known that the spontaneous final height obtained differs according to the type of aberration specific to TS [1,4,5]. Recombinant human growth hormone (rhGH) is commonly used in the long-term treatment of children with TS. The aim of this therapy is to achieve a higher growth and, therefore, improve the self-esteem, social status, and overall quality of life of girls with TS. The rhGH therapy may improve the final height of TS children up to 1.28 SD in comparison to their final height prognosis [6]. This standard growth-promoting treatment is carried out with empirical and fixed doses of rhGH, adjusted to body weight or surface, resulting in a variable growth response [2,3]. The addition of genetic data may improve mathematical models to predict the response to rhGH therapy and consequently allow a more personalized rhGH treatment strategy [7].

TS girls have different response to rhGH therapy, which may be triggered by heterogeneous groups of X chromosomal aberrations underlying TS clinical manifestation. Unfortunately, therapy with the use of rhGH is still expensive; therefore, in order to reduce the costs and raise its effectiveness, a special and very individual approach is required. Thus, the aim of this study was to assess the influence of karyotype abnormalities associated with the aberration of the X chromosome on growth improvement in the TS patients treated with rhGH.

## 2. Materials and Methods

This study was approved by the Bioethics Committee of the Medical University of Lodz, Poland (RNN/856/13/KB).

To verify our hypothesis, the retrospective cohort study was conducted in 57 Caucasian origin patients with TS (mean initial age ± standard deviation; 9.7 ± 3.1 years), who underwent rhGH therapy (somatropin) for at least 3 years and were followed up during regular visits in the outpatient clinic of the Department of Endocrinology and Metabolic Diseases of the Polish Mother’s Memorial Hospital Research Institute in Lodz, Poland. All patients enrolled to the study were diagnosed with TS between 2000 and 2009 on the basis of peripheral blood karyotyping. The patients were treated according to the rules of the therapeutic program, approved and supported financially by the Polish Ministry of Health. The patients who presented chronic systemic illnesses, or hypothyroidism were either excluded from the study or treated until achieving full alignment of their disorders before the implementation of the rhGH therapy. In the initially analyzed group of TS girls, spontaneous puberty was noted in one-with 45X karyotype and two-with mosaicism. We excluded them from the study so that the puberty did not affect the analyzed parameters. The remaining girls had no signs of central puberty at the time of analysis. Thus, the group was homogeneous. During the follow-up, none of the TS patients were treated with estrogen preparations. The dosage of rhGH varied from 1.0 to 1.25 IU/kg/week (with a mean dose of 1.04 ± 0.09 IU/kg/week), and the hormone was administered subcutaneously daily, before sleep. All of the enrolled subjects visited the outpatient clinic for follow-up every 3 months, and their height and body mass were measured. Height was checked with the use of a Harpenden stadiometer and marked to the nearest 0.1 cm. Body mass was measured with an electronic weighing scale to the nearest 0.1 kg. That was followed by calculation of the body mass index standard deviation score for chronological age. Every 6 months, the concentrations of TSH, free T4, insulin-like growth factor 1 (IGF 1), insulin-like growth factor building protein 3 (IGFBP3) and glycated hemoglobin (HbA1c) were assessed. Bone age (BA) assessment was performed every 12 months and interpreted according to the Greulich and Pyle method. Target height (TH) was calculated as the mean of parental height minus 6.5 cm. The TS patient’s height standard deviation score (H SDS) was calculated before the start of the therapy (H0 SDS) and after 1, 2, and 3 years of treatment (H1 SDS, H2 SDS, and H3 SDS, respectively). H SDS values were used to evaluate the responses to rhGH treatment, after calculating its changes in each of the previous years (delta H SDS). The height SDS was calculated according to the 2001 Polish reference charts for children and adolescents [8], as well as according to reference charts for untreated TS girls [9]. In summary, TH and TH SDS, as well as H SDS and delta between values of H SDS in subsequent years of rhGH therapy, were used to show the outcomes of the 3 year treatment.

Karyotype analysis was carried out with the use of peripheral blood lymphocytes. A detailed microscopic and computed analysis of the karyotype was performed with a resolution of 550 stripes. A polymerase chain reaction (PCR) with the use of starters specific to the intervals of the deletion map of Y chromosome was made to verify the presence of its fragments. Patients were divided into four study groups, according to their structured or numerical abnormalities of the karyotype as X monosomy (*n* = 35), isochromosome (*n* = 11), marker chromosome (*n* = 5), and X-mosaicism (*n* = 6). It is to be stressed that mosaic patients with the presence of the X long-arm isochromosome were classified to isochromosome group. 

The data were analyzed using Statistica 10.0 software (StatSoft, Inc.; Tulsa, OK, USA). The continuous variables were expressed as means ± standard deviation for normally distributed variables. The Kruskal–Wallis test was used to evaluate differences among groups. Shapiro–Wilk’s test was used to test the distribution of the variables. Annually measured parameters, associated with height changes, were compared using Student’s *t*-test (or nonparametric Mann–Whitney U-test) and chi^2^ test. Multiple regression analysis was used to evaluate the relationship between the height outcome and independent factors of clinical parameters. Correlations were evaluated using the Pearson’s test or Spearman’s rank correlation coefficient (*r*) test, depending on normality of distribution. A *p*-value <0.05 was considered statistically significant.

## 3. Results

All of the enrolled patients were treated with rhGH for more than 3 years and observed regularly during the follow-up visits in the outpatient clinic every 3 months. Under the follow-up observation, no adverse events such as type 2 diabetes or slipped capital epiphyses were observed in any of the subjects.

The frequency of karyotype abnormalities of TS subjects who participated in the study is shown in Table 1.

The monosomy of X chromosome was the most common chromosome aberration in the study cohort and was observed in 61.4% of the girls (*n* = 35). Baseline auxological data at the time of first rhGH administration according to the karyotype abnormalities are presented in Table 2.

It should be stressed that the statistical analysis demonstrated no significant differences in the baseline age, baseline height SDS (H0 SDS), target height, and initial dosage of rhGH in selected groups of karyotype abnormalities. Despite the fact that the patients with marker chromosome or mosaicism tended to be older at the time of enrollment to rhGH therapy, their height deficits at that time were similar to the other groups. In each subject in those groups, the subsequent initiation of treatment was associated with a lack of dysmorphic characteristics and the later visualization of growth deficits. Additionally, in the group of patients with TS and marker chromosome, the delay of rhGH therapy was related to a necessity to perform gonadectomy in all children before starting treatment.

**Figure 1:**
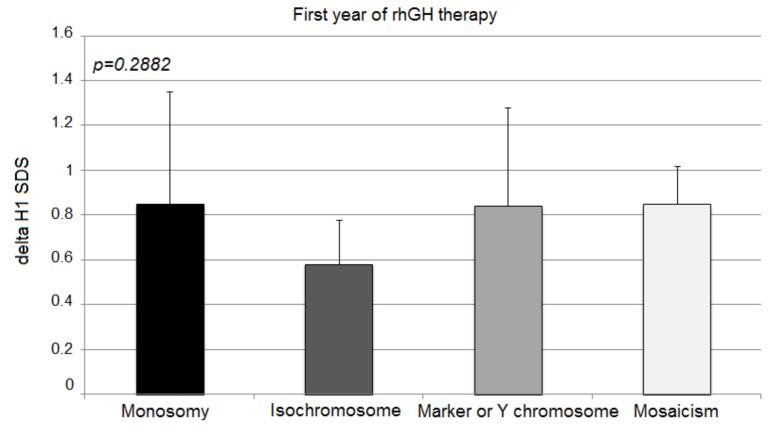
Growth response to rhGH during the first year of therapy. H1 SDS, height standard deviation score after 1 year of therapy. The ANOVA test was applied (*p* > 0.05).

During the first year of the therapy, all groups responded well to the rhGH therapy, and there were no statistically significant differences in delta H1 SDS in the first year of rhGH therapy among groups: monosomy 0.85 ± 0.55 SD; isochromosome 0.58 ± 0.20 SD; marker chromosome 0.84 ± 0.44 SD; mosaicism 0.85 ± 0.17 SD; *p* = 0.2882) (Figure 1).

TS patients with isochromosome tended to show a poorer response during the first year of rhGH therapy; however, statistical significance was not recorded. The second and third years showed the greatest difference in the effectiveness of the therapy.

**Figure 2:**
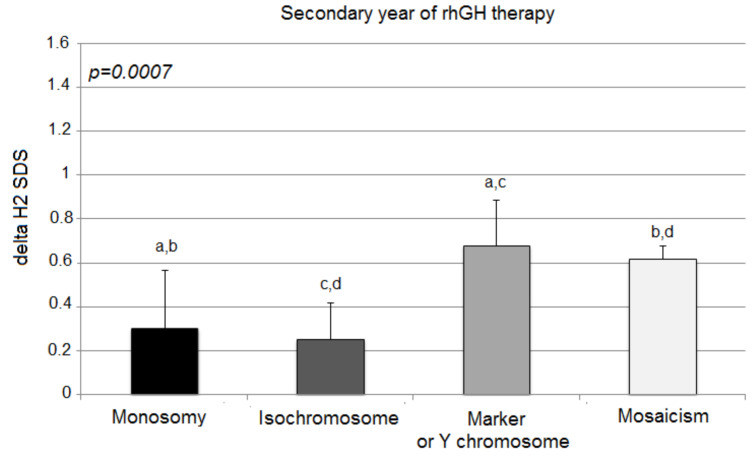
Growth response to rhGH during the second year of therapy. H2 SDS, height standard deviation score after 2 years of therapy; the ANOVA test was applied (*p* < 0.001). Values (bars) marked with the same letters are significantly different (Dunn’s multiple comparisons test): a—*p* < 0.04; b—*p* < 0.032; c—*p* < 0.015; d—*p* < 0.012. The groups designed with the same letter different significantly from each other: a—*p* = 0.040; b—*p* = 0.032; c—*p* = 0.015; d—*p* = 0.012.

The second year’s response expressed with delta H2 SDS deteriorated significantly in patients with X monosomy and isochromosome in comparison to the other two groups (monosomy 0.30 ± 0.27; isochromosome 0.25 ± 0.17; marker chromosome 0.68 ± 0.21; mosaicism 0.62 ± 0.06; *p* = 0.0007) (Figure 2).

In comparison to the first year of rhGH therapy, a strong deceleration of delta H2 SDS was observed in children with X-monosomy and isochromosome (0.85 ± 0.55 vs. 0.30 ± 0.27; *p* < 0.0001; 0.58 ± 0.20 vs. 0.25 ± 0.17; *p* = 0.0004; respectively). Moreover, the mosaicism group showed a slightly statistically significant slowdown of growth in the second year, albeit not as strong as in the previous groups (0.85 ± 0.17 vs. 0.62 ± 0.06; *p* = 0.0108). Patients with marker chromosome sustained their good response to rhGH after the second year of treatment in comparison to delta H1 SDS (0.84 ± 0.44 vs. 0.68 ± 0.21; *p* = 0.501). 

**Figure 3:**
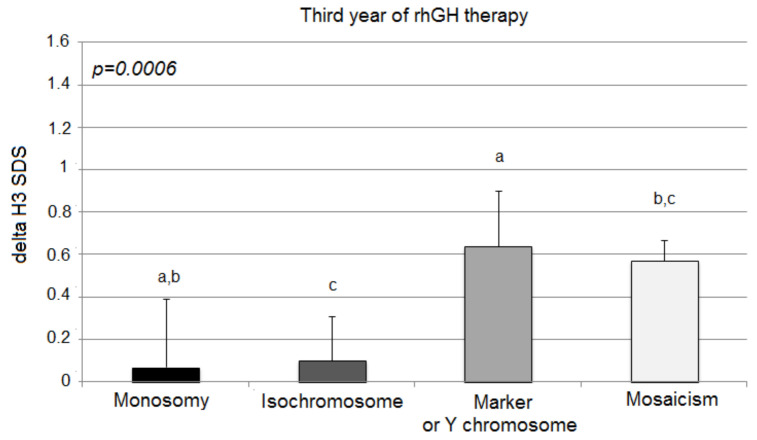
Growth response to rhGH during the third year of therapy. H3 SDS, height standard deviation score after 3 years of therapy; the ANOVA test was applied (*p* < 0.001). Values (bars) marked with the same letters are significantly different (Dunn’s multiple comparisons test): a—*p* < 0.018; b—*p* < 0.011; c—*p* < 0.043. The groups designed with the same letter different significantly from each other: a—*p* = 0.018; b—*p* = 0.010; c—*p* = 0.043.

The third year of the therapy once again showed statistically significant differences in growth among the analyzed groups, expressed with delta H3 SDS (monosomy 0.07 ± 0.32; isochromosome 0.10 ± 0.21; marker chromosome 0.64 ± 0.26; mosaicism 0.57 ± 0.10; *p* = 0.0006) (Figure 3).

However, in comparison to delta H2 SDS, delta H3 SDS deteriorated significantly only in children with X-monosomy (0.30 ± 0.27 vs. 0.07 ± 0.32; *p* = 0.003). The other groups of patients (isochromosome, marker chromosome, and mosaicism aberrations) tended to sustain their response to the rhGH therapy (delta H3 SDS vs. delta H2 SDS; 0.10 ± 0.21 vs. 0.25 ± 0.17; *p* = 0.101; 0.64 ± 0.26 vs. 0.68 ± 0.21; *p* = 0.789; 0.57 ± 0.10 vs. 0.62 ± 0.06; *p* = 0.353, respectively).

It has to be stressed that the 3 year therapy with the use of rhGH improved the height of all groups of patients (Table 3) in comparison to their TH SDS, although the best outcomes were achieved in patients with marker chromosome or Y chromosome and patients with X-mosaicism (Table 4).

## 4. Discussion

Turner syndrome, as a condition with similar clinical manifestations but various genetic bases, may happen to generate many difficulties in both the diagnosis and the treatment process. Currently, therapy with the use of rhGH, which has been approved worldwide, brings new hope for TS patients. By improving the final height, the therapy greatly increases the quality of the patient’s life and their social status. However, it has commonly been observed that not all patients tend to respond equally to the administrated therapy [3,10].

The current guidelines offer a standard and not individualized dosage of the hormone for all of the patients, regardless of the karyotype underlying the cause of TS [3,11]. In our opinion, genetic differences may underlie the poorer or better response to the rhGH in different karyotype groups of patients, a problem which was examined in our study. 

Our results proved a statistically significant better response to the standard doses used in the therapy in the groups of patients with mosaicism and with the presence of marker chromosome or Y chromosome. On the other hand, patients with simple X-monosomy or isochromosome had poorer outcomes of the standard therapy.

The mechanism underlying the impaired height in TS and the results of our study, however, are not related to the lack of growth hormone in patients. It should be emphasized that, in some TS girls, growth hormone deficiency (GHD) is simultaneously observed [12], but none of our patients had GHD.

The etiology of this condition has been proven to be connected with the haploidity of the SHOX gene, located in the pseudoautosomal region of the short arm of the X chromosome (PAR1) [13,14]. The SHOX gene plays an important role as a transcription factor, controlling the growth of bones, as well as differentiation and maturation of the chondrocytes. Although, in healthy women, one copy of the X-chromosome undergoes inactivation, the SHOX gene is one of those inherited from both parents in two active copies. Two active copies of the SHOX gene determine the normal height. Haploidity of this gene is proven to cause the short stature of the patients. TS patients with the karyotype 45,X and patients with isochromosome of the long arm of X tend to have a shorter final height than patients with other types of chromosome abnormalities [4,12,15]. It may also result in a poorer response to the therapy, as shown in our study. This can be easily noticed, especially in comparison with the mosaicism patients of the 45,X/46,XX and 45,X/46,XY karyotype. Moreover, patients with deletions of the long arm of the X (46,X, Xq del) tend to have a final height similar to the normal population [3,4]. Those mechanisms are probably the basic ones influencing the outcomes of this study. Although we are aware of the small number of mosaic and marker chromosome patients included in the study and the difficulties this may cause in making any statistical conclusions, we find the results quite interesting and worthy of further studies. It should be emphasized that the small number of these patients correlates with the general frequency of such karyotypes in the population of TS children.

The SHOX gene and its haploidity is not the only mechanism underlying short stature and does not explain all the problems connected with impaired growth in girls with TS and the response to the therapy. It has been noticed that, in patients with a simple haploidity of the SHOX gene, there is no such growth deficit as in TS patients [15,16]. It is plausible that there are other genes of the short arm of X-chromosome, the lack of which may cause the growth delay and/or the poorer response to the therapy. It was also postulated that the aneuploidy itself may also be a reason for impaired growth [4]. All of these elements might have led to the obtained results showing a statistically poorer response of the patients with X-monosomy in comparison to the mosaic patients, as well as patients with marker chromosome or Y chromosome [15,16].

According to a very recent report, the growth velocity in girls with TS who undergo treatment with rhGH may also be connected to the polymorphisms of various genes that are not found on the X chromosome [17]. Two polymorphisms of two genes of chromosome 1 and 12 are thought to be connected with the response to the therapy with rhGH. These are the signaling molecule KRAS and the pituitary transcription factor LHX4, responsible for the differentiation and maturation of the pituitary gland. The differences in height according to the presence or absence of the alleles of those genes were more than 1 cm in the first year of the therapy. LHX4 was connected with a better response to the therapy [17].

Another possible factor influencing the response to the rhGH therapy and the final height reached by the patients is the SOCS2 gene and its polymorphisms, as reported in a Brazilian study conducted in 2014 [7]. SOCS2 is a suppressor of cytokine signaling, and it acts as an intracellular negative regulator of GH receptor signaling. According to the study, SOCS2 polymorphism has an influence on the adult height of children with TS after long-term rhGH therapy. Females with TS carrying at least one SOCS2 SNPrs37822415-C allele were proven to reach a 0.7 SD higher adult height than those who were homozygous for the T allele. 

However, as all of these genes are situated on the autosomal chromosomes, they should not influence the results of our study, assuming that all of the girls from all the karyotype groups were affected in the same way [4,7]. In 2020, Godoy-Molina et al. showed that there were no significant differences in adult height between TS women treated with GH (70%) and those not treated (30%), and that the median height was 150 cm a group of 70 TS women [5]. On the other hand, among women with TS treated with rhGH, not all show success in improving final height. Other factors that could influence the therapy and its results are the height of the parents and the therapy starting age. The best response to the therapy is proven to be correlated with the starting age, whereby the girls who begin the therapy earlier tend to grow better [18,19]. The mean birth length and weight of the body of the infants with TS is usually only a little lower than the mean length and weight of healthy newborns. Although an impaired growing process may already be noticed in the 12th to 18th month of life, many of the cases are unfortunately diagnosed only when the children are around 6 years old or even older [5], which makes it more difficult to implement the therapy at an early age and improve its efficacy. The mean growth velocity in untreated prepubertal patients with TS is below 4 cm per year. After the implementation of the therapy with rhGH, it is possible to obtain a considerable improvement in the growth velocity, which is easily observable, especially during the first year of the therapy, which was also confirmed by our results [3,20]. Japanese authors (2013) showed that the growth velocity in girls treated with rhGH during the first year of therapy was 8.15 cm per year, which made it twice the value for untreated patients with TS [21]. The consecutive years of therapy showed a decrease in the growth velocity. Thus, once again, similar results were obtained in our study [18,21].

The analysis presented in our article covers the period during which the program of treating TS girls with GH in Poland was just started. That is why the age for starting therapy was so late. Today, due to progress in the childhood diagnosis of TS, treatment is usually initiated much earlier, around the age of 4–6 years. Therefore, this is a unique and valuable group of girls. Moreover, in our department, estrogen therapy in girls with TS begins later at the age of 13–14, when the obtained height is satisfactory, and the bone age is not advanced. This allows for a growth spurt; hence, none of the presented patients were treated with E–P therapy. Many authors believe the first 2 years of treatment to be an indicator of response to rhGH therapy [22,23]. 

In conclusion, our investigation clearly showed that X-monosomy or the presence of isochromosome determines a poorer response during the second and third years of rhGH therapy in children with TS. On the other hand, the best response to the rhGH therapy during the second and third years was observed in TS patients with X-mosaicism and marker chromosome or Y chromosome. It is important to remember that the observation of the growth velocity in all of the groups is not yet finished. Therefore, the final effects of the therapy might still improve, especially in younger patients. However, it should be taken into account that, in X-monosomy and isochromosome groups, the improvement is not expected to be significantly greater.

It is well known that the first and second years of treatment are usually a catch-up period, and the subsequent years represent a period of stabilization of the effect. Due to the fact that, in girls with TS, in addition to rhGH, the growth-promoting effect depends on the initiation of E–P therapy, we used the prepubertal period for our analysis, and we closed it at the stage just before starting E–P therapy [22,23]. 

It is also worth discussing that, so far, no significant complications of rhGH treatment have been observed in girls with TS, despite the fact that it is not a substitution treatment, and that the benefits of rhGH treatment outweigh the possible complications [24]. According to the database from the Short Stature International Study, no serious complications (especially deaths) were found in 948 TS patients treated with GH for at least 5 years [25].

The results of the study might prove the need for a more individualized dosage of rhGH during the second and the third years of the therapy in TS patients with X-monosomy and isochromosomes. In our opinion, in some cases, the highest possible dose of rhGH could be used, which would still not increase the concentration of IGF-1 above the upper limit of range. Thus, our suggestion indeed applies to the possibility of increasing the doses of GH in the treatment of TS, based on HV and IGF-1 levels. This seems to depend on the karyotype, as shown in our study using recommended rhGH doses alone, where the effects were very different. We also do not suggest discontinuation of treatment in some cases, although the data from the literature indicate that some patients achieve a normal final height despite a lack of treatment. It now appears that the benefits of treating girls with TS using rhGH in order to ensure nondiscriminatory population growth are undeniable. Further studies should be performed in a larger cohort to confirm our observations and to verify whether there are differences between the escalated dosage of rhGH and the efficacy of the therapy in the groups with a poorer response. The specific genetic background and other factors influencing the efficacy of the therapy should also be examined.

## Figures and Tables

**Table 1:** Karyotypes abnormalities in patients with TS.

Monosomy (*n* = 35)	Isochromosome (*n* = 11)	Marker Chromosome or Y Chromosome (*n* = 5)	Mosaicism (*n* = 6)
45,X	46,X,i(Xq)	45,X/46,X,+mar	45,X/46,XX
	46,X,i(X)(q10)	45,X/47,XYY	45,X/46,X,+r
	46,X,i(X),(q10),inv(9)(p11q13)		45,X/46,X,del(X)(q12-13)
	46,X,idic(X)(p11)		45,X/46,X,del(X)(q22)
	45,X/46,X,i(X)(q10)		
	45,X/46,X,i(Xq)		

**Table 2:** Baseline clinical characteristics of TS patients undergoing rhGH therapy.

	Monosomy (*n* = 35)	Isochromosome (*n* = 11)	Marker Chromosome or Y Chromosome (*n* = 5)	Mosaicism (*n* = 6)	*p*-Value
Initial age, years	9.05 ± 3.11	9.79 ± 2.64	12.47 ± 2.46	11.25 ± 2.83	0.0920
TH, cm	161.80 ± 5.46	160.16 ± 5.40	158.52 ± 5.97	163.52 ± 5.78	0.5973
TH SDS	−0.70 ± 0.91	−0.97 ± 0.90	−1.25 ± 0.99	−0.41 ± 0.96	0.5973
H0, cm	116.96 ± 15.03	117.71 ± 11.72	130.30 ± 9.86	129.00 ± 13.78	0.1159
H0 SDS	−3.27 ± 0.99	−3.92 ± 0.77	−3.74 ± 1.31	−2.89 ± 0.98	0.1399
H0 SDS*	+0.52 ± 0.9	+0.1 ± 0.75	+0.5 ± 1.01	+1.2 ± 1.21	0.1340
BMI SDS	−1.78 ± 1.52	−2.56 ± 1.38	−1.89 ± 1.81	−0.75 ± 1.70	0.3861
Initial dosage of rhGH, IU/kg/week	1.04 ± 0.08	0.99 ± 0.09	1.09 ± 0.05	1.09 ± 0.16	0.1095

Data are presented as means ± standard deviation; TH, target height; TH SDS, target height standard deviation score; H0, initial height; H0 SDS, initial height standard deviation score; H0 SDS*, initial height standard deviation score according to untreated TS centile charts; BMI SDS, body mass index standard deviation score; rhGH, recombinant human growth hormone.

**Table 3:** Mean values (±SD) of the height deficiency in particular groups of girls with TS before treatment and after 1, 2, and 3 years of rhGH therapy.

	Monosomy (*n* = 35)	Isochromosome (*n* = 11)	Marker Chromosome or Y Chromosome (*n* = 5)	Mosaicism (*n* = 6)	*p*-Value
H0 SDS	−3.27 ± 1.00	−3.92 ± 0.77	−3.74 ± 1.31	−2.89 ± 0.98	0.1399
H1 SDS	−2.42 ± 0.86	−3.34 ± 0.78	−2.90 ± 0.97	−2.04 ± 0.83	0.0094
H2 SDS	−2.12 ± 0.87	−3.10 ± 0.71	−2.22 ± 0.86	−1.42 ± 0.83	0.0022
H3 SDS	−1.97 ± 0.91	−3.05 ± 0.58	−1.79 ± 0.62	−0.85 ± 0.86	0.0007
*p*-Value	<0.0001	0.0114	0.0499	0.0211	

Data are presented as the mean ± standard deviation; H0 SDS, initial height standard deviation score; H1 SDS, height standard deviation score after 1 year of therapy; H2 SDS, height standard deviation score after 2 years of therapy; H3 SDS, height standard deviation score after 3 years of therapy.

**Table 4:** Comparison of the growth catch-up in reference to target height.

	Monosomy (*n* = 35)	Isochromosome (*n* = 11)	Marker Chromosome or Y Chromosome (*n* = 5)	Mosaicism (*n* = 6)	*p*-Value
TH SDS–H0 SDS	2.61 ± 1.01	2.95 ± 0.93	3.10 ± 1.53	2.48 ± 1.59	0.8129
TH SDS–H3 SDS	1.34 ± 0.95	2.21 ± 0.75	0.65 ± 0.97	0.15 ± 1.33	0.0056
Improvement	1.34 ± 0.88	0.90 ± 0.28	2.66 ± 1.13	2.02 ± 0.27	0.0057

## Data Availability

The datasets used and/or analyzed within the framework of this study are available from the corresponding author on reasonable request.
